# Immunotherapeutic Agents for Intratumoral Immunotherapy

**DOI:** 10.3390/vaccines11111717

**Published:** 2023-11-14

**Authors:** Chih-Rong Shyr, Lang-Chi Liu, Hui-Shan Chien, Chi-Ping Huang

**Affiliations:** 1 Department of Medical Laboratory Science and Biotechnology, China Medical University, Taichung 404328, Taiwan; chshyr@mail.cmu.edu.tw (C.-R.S.); u110073202@cmu.edu.tw (H.-S.C.); 2 eXCELL Biotherapeutics Inc., Taichung 404328, Taiwan; 3 Department of Medicine, Department of Surgery, College of Medicine, China Medical University and Hospital, Taichung 404328, Taiwan; d7495@mail.cmu.edu.tw; 4 Department of Medicine, Urology Division, China Medical University and Hospital, Taichung 404328, Taiwan

**Keywords:** immunotherapeutic, intratumoral, antitumor immunity, body, neoantigen, xenoantigen

## Abstract

Immunotherapy using systemic immune checkpoint inhibitors (ICI) and chimeric antigen receptor (CAR) T cells has revolutionized cancer treatment, but it only benefits a subset of patients. Systemic immunotherapies cause severe autoimmune toxicities and cytokine storms. Immune-related adverse events (irAEs) plus the immunosuppressive tumor microenvironment (TME) have been linked to the inefficacy of systemic immunotherapy. Intratumoral immunotherapy that increases immunotherapeutic agent bioavailability inside tumors could enhance the efficacy of immunotherapies and reduce systemic toxicities. In preclinical and clinical studies, intratumoral administration of immunostimulatory agents from small molecules to xenogeneic cells has demonstrated antitumor effects not only on the injected tumors but also against noninjected lesions. Herein, we review and discuss the results of these approaches in preclinical models and clinical trials to build the landscape of intratumoral immunotherapeutic agents and we describe how they stimulate the body’s immune system to trigger antitumor immunity as well as the challenges in clinical practice. Systemic and intratumoral combination immunotherapy would make the best use of the body’s immune system to treat cancers. Combining precision medicine and immunotherapy in cancer treatment would treat both the mutated targets in tumors and the weakened body’s immune system simultaneously, exerting maximum effects of the medical intervention.

## 1. Introduction

Cancer is a major public health problem worldwide, which causes approximately 10 million deaths each year, mostly due to advanced cancers when tumors metastasize or are inoperable with surgery. At this stage, patients have mostly been treated with conventional therapies such as surgery, radiotherapy, chemotherapy and targeted therapy as well as the combination of these treatments, but eventually, all these treatments fail, and patients die. Because these modalities use physical or chemical methods to treat tumors cells, either the physical forces are too strong and damage normal tissues or the chemical strength is too toxic for normal cells, and the body eventually succumbs to cancer and dies. Cancer deaths result from infection, organ failure, infarction, hemorrhage and cardiovascular diseases because of failing bodies [1,2]. The body’s immune system is able to detect and destroy transformed tumor cells by cytotoxic innate and adaptive immune cells, but the tumor cells evolve different mechanisms that help them to escape immune attacks [3]. Thus, the goal of cancer immunotherapy is to repress tumor cell escape mechanisms and to reactivate antitumor immunity.

### 1.1. Systemic Cancer Immunotherapy

Conventional therapies like surgery, radiotherapy, chemotherapy and targeted therapy all target tumors to treat cancer. Cancer interacts with the body’s immune system that involves multiple factors, diverse cell lineages and the interactions among them. To stimulate antitumor immunity, proper immune responses need to be driven out to enhance tumor eradication by the body’s immune system. The understanding of immune system control in tumor growth and development has increased significantly in recent years, resulting in the development of cancer immunotherapy that has changed the outcomes and extended the survival of advanced cancer patients. This recent re-emergence of immunotherapy has made a breakthrough in saving cancer patients’ lives by targeting the body’s immune system to treat cancer. Cancer immunotherapy harnesses the body’s immune system to treat cancers by eliciting the innate and adaptive immune responses to eradicate tumor cells; by using the body’s own immune system to fight off cancer cells, the target is the body’s immune system rather than the tumor cells. Among cancer immunotherapy approaches, one approach is to increase antitumor T-cell responses, either by blocking inhibitory pathways with immune checkpoint inhibitors (ICIs) or by targeting stimulatory pathways like chimeric antigen receptor T cells [4]. ICIs are checkpoint blockade monoclonal antibodies that target inhibitory immune checkpoint proteins like programmed death (PD) receptor 1 (PD-1), PD ligand 1 (PD-L1) and the cytotoxic T-lymphocyte-associated protein 4 (CTLA-4). ICIs have achieved significant clinical successes in extending overall survival with durable disease control for advanced cancer patients across different tumor types. However, only a subset of cancer patients (20–40%) have benefited from this treatment [5]. In terms of the safety of ICI drugs, the incidence of irAEs ranges between 54% and 76% for all adverse events and grade 3 or 4 irAEs comprise 31% of all irAEs for CTLA-4 inhibitors and 10% for PD-1 inhibitors [6,7]. Adoptive immune cell therapy with T cells carrying chimeric antigen receptors (CARs) demonstrates strong efficacy against hematological malignancies, but has not been shown to be effective in solid tumors.

All of the above treatments are administered intravenously and rely on systemic biodistribution to deliver the therapeutic agents into tumors in order to exert their effects, which could be hindered by the immunosuppressive tumor microenvironment (TME). The TME includes stromal cells and immune cells such as natural killer (NK) cells, T cells and a variety of myeloid cells, including myeloid-derived suppressor cells (MDSCs), dendritic cells (DCs) and macrophages, and surrounding tumor cells as well as the extracellular matrix scaffolds supporting the cells. The immunosuppressive cold tumor microenvironment lacks infiltrating antitumor T cells to eradicate tumor cells, but fills with MDSCs, M2-polarized macrophages and regulatory CD4+ T cells (Treg) as well as immunosuppressive cytokines like TGF-β, and is linked to the failure of systemic immunotherapy [8]. In addition, systemic agents cause systemic toxicities, such as ICI mAbs with potentially irAEs and CAR T cells with cytokine release syndrome, which are all life threatening [9]. In one case report, a CAR T-cell therapy was used that recognized ERBB2 with CD28, 4-1BB and CD3ζ signaling moieties to treat a patient with colon cancer that had metastasized to the lungs and liver and was resistant to multiple standard therapies. The patient experienced respiratory distress 15 min after receiving 10^10^ cells intravenously; despite intensive medical intervention, after 5 days treatment, the patient still passed away [10].

### 1.2. Locoregional Intratumoral Immunotherapy

To maximize the benefits of cancer immunotherapy and to lessen systemic toxicities, in recent years, locoregional intratumoral immunotherapy has been studied to complement the deficiency of systemic immunotherapy. However, a tumor-directed approach to induce immune responses to attack tumors had been investigated more than a century ago by Dr. William B. Coley, regarded as the father of cancer immunotherapy. Considered to be the origin of modern cancer immunotherapy, by injecting bacteria toxins (a cocktail of heat-killed Streptococcus pyogenes and Serratia marcescens) intratumorally with the purpose to develop erysipelas to stimulate the immune system, Dr. Coley demonstrated the efficacy of this treatment in patients with osteosarcoma [11,12]. Intratumoral immunotherapy strategies that involve direct injection of different immune-stimulating agents into tumor sites recapitulate Dr. Coley’s idea that activating the body’s immune system locally could eradicate tumors systemically. This approach could largely reduce systemic toxicities and adverse events since the main immune responses occur locally and the approach could be combined with other systemic therapies without adding more toxicities [13,14]. Moreover, the key step in the cancer immunity cycle to eliminate tumor cells is the recruitment of antitumor effector T cells into tumor sites, and thus local immune activation inside tumors to attract effector T cells is very critical. This critical event could be achieved through intratumorally administered immunotherapeutic agents that trigger various immune reactions inside tumors based on their immunological properties. In addition, the circulating effector T cells could once again travel to noninjected tumor sites to execute anenestic or abscopal effects if there are metastatic lesions [15]. Compared with other local therapeutic modalities like surgery and radiotherapy, because of its minimally invasive procedure through needle injection under image guide, intratumoral immunotherapy could still be performed in unresectable or medically inoperable tumors. Additionally, because it targets the body’s immune system, intratumoral immunotherapy does not cause direct normal tissue damage such as with resection or radiation.

## 2. Classes of Intratumoral Immunotherapeutic Agents in Preclinical and Clinical Studies

**Figure 1:**
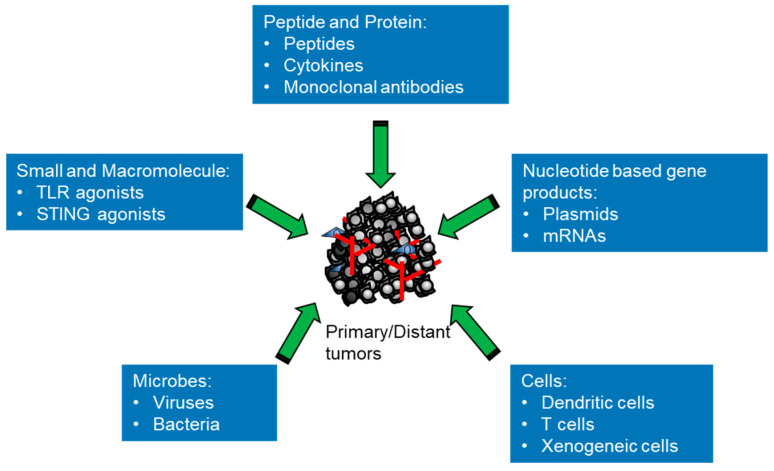
The classes of immunotherapeutic agents for intratumoral immunotherapy. Classification of the different types of immunotherapeutic agents from small molecules to cells used for intratumoral immunotherapy that induce the immune responses in the body to revive and direct antitumor immunity for the eradication of tumor cells.

**Figure 2:**
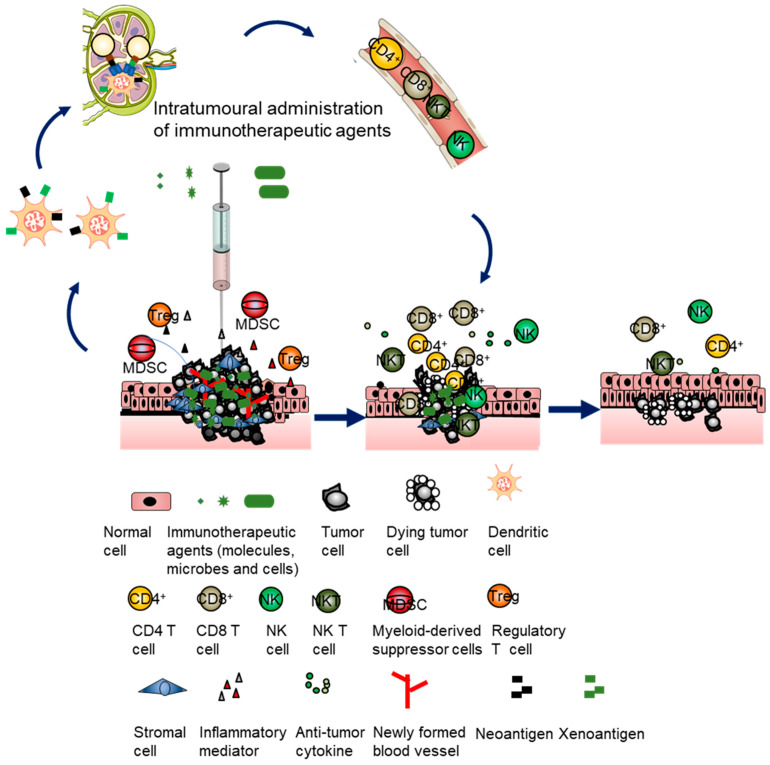
Intratumoral immunotherapy involves direct injection of immunotherapeutic agents into tumors. The immunosuppressive tumor microenvironment of an injected tumor is stimulated to turn the immunosuppressive state into an immunostimulatory state by using the injected agents, involving innate and adaptive immunity depending on the class of agents. The immune-active tumor microenvironment promotes recruitment of NK and DC cells as well as recognition of tumor antigens by DCs. The tumor antigen-loaded DCs present tumor antigens to T cells, triggering the generation of polyclonal effector and memory T cells. Then, the primed T cells circulate systemically to infiltrate into injected tumors and noninjected tumors to achieve durable and global antitumor immune responses to eliminate and contain tumor cells.

The therapeutic principle of intratumoral immunotherapy is to elicit local immune responses to generate both local and systemic antitumor effects to eradicate all tumor cells in the body, bringing durable clinical benefits. There are different classes of agents that can potentially elicit various antitumor immune responses inside the body to react to the broad tumor heterogeneity (Figure 1 and Table 1). From small molecules, macromolecules and genetic materials to living therapeutics, all types of substances that stimulate the immune system with different actions can be tested with direct administration into tumors for evaluating their capacity to either prime or boost immune responses. This approach provides a versatile platform for modulating key components of the antitumor immune response, such as activation of T cells, B cells and macrophages [16]. There are a number of intratumoral immunotherapies with differing mechanisms of action that have been studied in preclinical models and some have been tested successfully in clinical trials, showing promising results. In this review, in particular, we focus on new developments concerning different types of immunotherapeutic agents used for intratumoral immunotherapy. We briefly summarize the signaling and outline the mechanisms exerted by these agents (Figure 2). According to their sizes and attributes, intratumoral immunotherapeutic agents can be divided into different classes as follows: small and macromolecules (e.g., TLR agonists); proteins (e.g., peptides, cytokines or mAbs); nucleotide-based gene products (e.g., plasmids or mRNAs); viruses (e.g., oncolytic viruses or viral vectors for gene therapy); bacteria (e.g., *Salmonella typhimurium*); and cells (e.g., autologous/allogeneic DCs, autologous/allogeneic CAR-T or xenogeneic tissue cells).

### 2.1. Small and Macromolecules

As the essential components of the body’s innate immune system, the mammalian Toll-like receptors (TLRs) are germline-encoded receptors expressed in cells as sensors to respond to various distinct structural moieties present in microbes known as pathogen-associated molecular patterns (PAMPs) or endogenous damage-associated molecular patterns (DAMPs) in injured cells. PAMP/DAMP-TLR bindings elicit the secretion of proinflammatory cytokines and the subsequent differentiation and maturation of antigen-presenting cells (APCs) that target invading microbes or damaged cells with adaptive immune responses. Different types of TLRs react to particular PAMPs in order to recognize and respond to invading microbial pathogens. Lipoteichoic acid and peptidoglycan of bacterial components stimulate TLR2/TLR6 heterodimer, double-stranded RNAs from viruses activate TLR3, lipopolysaccharides (LPS) from Gram-negative bacteria stimulate TLR4, guanosine- or uridine-rich single-stranded RNAs from viruses activate TLR7/8 and viral DNAs rich in unmethylated CpG motifs stimulate TLR9 [68]. In addition to TLRs to trigger protective innate and adaptive immune responses, the cyclic GMP–AMP synthase (cGAS)-stimulator of the interferon (IFN) genes (STING) pathway detects double-stranded DNA (dsDNA), a product resulting from viral or bacterial infection or severe tissue damage through the allosteric binding of the dsDNA to the nucleotide cyclase enzyme cGAS, which subsequently generates cyclic guanosine monophosphate–adenosine monophosphate (cGAMP) dinucleotides to activate STING for the induction of innate immune reactions by inducing transcription of IFNs and numerous IFN-stimulated genes as well as NF-κB-mediated expression of proinflammatory cytokines genes [69,70].

Imiquimod is a small-molecule immunomodulatory compound that exerts its biological efficacy through agonistic stimulation of TLR7 that recognizes single-strand RNAs (ssRNAs) with viral features in immune cells. In a murine mesothelioma model, locally delivered imiquimod in combination with systemic anti-CD40 immunotherapy not only significantly enhanced the local antitumor response with 30% complete resolution, but also inhibited distal tumor growth [17]. The intratumoral administered STING agonist MIW815 (ADU-S100) has been shown to activate immune reactions but with limited antitumor activity in monotherapy and combined therapy with spartalizumab (PDR001), a humanized IgG4 antibody against PD-1; in advanced solid tumor or lymphoma, patients still showed minimal antitumor responses [18]. The TLR4 agonist G100 is a fully synthetic analogue of bacterial lipopolysaccharides (LPS) and has been tested intratumorally in patients with Merkel cell carcinoma; because TLR4 is involved in septic shock, systemic administration of its agonist could be dangerous. The results showed that intratumoral G100 was feasible and could be safely administered both in neoadjuvant and metastatic settings with encouraging clinical activity [71]. Intratumoral G100 with the addition of pembrolizumab as well has been investigated in a phase I/II trial involving patients with follicular lymphoma and, when adding pembrolizumab in treatment, G100 resulted in an overall response rate of 33.3% and abscopal tumor regression in 72.2% of patients with limited toxicities, demonstrating the potential for the combined treatment of the intratumoral TLR4 agonist with other agents to produce immune-mediated reactions in follicular lymphoma [19].

CpG oligodeoxynucleotides are TLR9 agonists. Intratumoral CpG oligodeoxynucleotide treatment with local low-dose radiotherapy in patients with low-grade B-cell lymphoma induced local and systemic antilymphoma clinical responses at the irradiated tumor site, regression of distant, nonirradiated sites of lymphoma in an immune-mediated response manner with a shorter time to disease progression and was well tolerated [72]. Intratumoral CpG oligodeoxynucleotide therapy was, once again, explored in metastatic solid tumor models as neoadjuvant intratumoral immunotherapy and with the addition of systemic anti-PD-1 Abs. The combination of intratumoral CpG oligodeoxynucleotides and systemic anti-PD-1 treatment increased survival due to their synergistic inhibitory effect on tumor-associated macrophages [20]. Vidutolimod is a virus-like particle containing a TLR9 agonist, i.e., CpG-A oligonucleotide, which activates plasmacytoid dendritic cells (pDCs) of the innate immune system to stimulate stronger adaptive immune responses. Intratumoral vidutolimod in combination with atezolizumab with and without radiation therapy was investigated in patients with PD-1- or PD-L1-resistant advanced NSCLC. The results showed modest pharmacodynamic and clinical activities in this heavily pretreated patient population and demonstrated that the procedure to intratumorally inject vidutolimod into the visceral lesions could be safe to practice [21].

Other macromolecules like glycolipids with α-gal (alα1-3Galβ1-4GlcNAc-R) epitopes (α-gal glycolipids) have also been explored as an intratumoral immunotherapeutic agent. After intratumoral α-gal glycolipid injection, α-gal glycolipids insert into tumor cell membranes and α-gal is bound by anti-Gal, the most abundant natural antibody in humans, constituting 1% of immunoglobulins; the binding activates complements and cleavage peptides C5a and C3a, and then recruits inflammatory cells and APCs into the treated lesion, converting tumor cells into an autologous tumor-associated antigen vaccine. A preclinical study has demonstrated intratumoral α-gal glycolipids treatment could prevent the development of metastases at distant sites and could defend against tumor rechallenge in treated mice [73]. Intratumoral injection of α-gal glycolipids has been tested in patients with advanced solid tumors, and the results indicated that intratumoral administration of α-gal glycolipids could be safely performed and could generate a protective antitumor immune response [22].

### 2.2. Peptides and Proteins

Peptides such as hormones consist of short chains of amino acids and proteins like cytokines, and antibodies (Abs) are macromolecules made up of multiple peptide subunits. Both are naturally occurring large molecules encoded by multiple genes in the body to perform different physiologic functions from metabolism to immunity. Peptides have been used as drugs because of their functions as hormones and neurotransmitters or in antimicrobial activities [74,75]. Cytokines are small, secreted proteins released from cells for interactions and communications between cells. Cytokines can be divided into proinflammatory cytokines and anti-inflammatory cytokines from immune cells or non-immune cells, which interact to keep the immune system in check and maintain the body in homeostasis. They are involved in controlling cancer, infections and other diseases. Abs are glycoproteins belonging to the immunoglobulin superfamily that are produced by B lymphocytes and involved in humoral immunity. As therapeutics, Abs could be used as stimulating agonistic Abs to activate immune signaling pathways and as blocking antagonistic Abs to inhibit their binding targets.

Oncolytic peptides have been studied for their actions as intratumoral immunotherapies. The oncolytic peptide LTX-315 (ruxotemitide) was designed based on the structure of bovine lactoferricin and has been studied as an intratumoral treatment in a mouse B16 melanoma model. Intratumoral administration of LTX-315 has been shown to cause complete tumor regression in the majority of mice and the recruitment of immune cells into the tumor parenchyma was found in LTX-315-treated mice [76]. A phase I dose-escalating study of intratumoral LTX-315 treatment tested in patients with advanced solid tumors showed that LTX-315 was safe with activities including shrinking the tumors, inducing CD8+ T-cell infiltration into the tumor microenvironment and expanding tumor-associated T-cell clones [23].

Recombinant cytokines have been applied in clinics to treat cancers, such as IFN alpha for follicular lymphoma, hairy cell leukemia and melanoma [77] and IL-2 for renal cell cancer and melanoma [78,79]. Systemic cytokine monotherapy requires administration of large quantities of cytokines to achieve a sufficient therapeutic effect, which causes severe toxicities. Thus, locoregional therapies that directly deliver immune-stimulatory cytokines to tumors have been proposed to increase the efficacy of cytokines, but reduce systemic toxicities. Repeated intratumorally injected IL-2 has mediated tumor regression through an endogenous, tumor-specific in vivo CTL response and reduced vasculature in a murine model of mesothelioma [80]. Local intratumoral injection of IL-2 after radiotherapy not only shrunk the irradiated tumor, but also inhibited distant metastasis development located outside the radiotherapy field in a Balb/c mouse model of simultaneous subcutaneous tumor and liver metastasis of the colon [81]. Intratumoral IL-2 in patients with stage III melanoma with cutaneous metastasis without lymph node involvement and stage IV melanoma with soft-tissue metastasis without visceral involvement demonstrated unexpected favorable survival rates, and the treatment was associated with increased complete and partial responses in subsequent chemotherapies [24]. Examination of intralesional injections of IFNα-2a in the treatment of basal cell carcinoma has revealed that eleven lesions (55%) showed complete clinical and dermatopathological remission, six lesions (30%) were found to be in partial remission, two lesions (10%) exhibited no response with low recurrence rates in long-term follow-up and no serious adverse effects were observed [25]. Intratumoral IFNα-2b has been investigated for the treatment of basal cell carcinomas and has been shown to be an alternative, effective, and cosmetically elegant treatment for basal cell carcinoma [26]. In a recent clinical trial, patients with stage IIIB, IIIC or IV melanoma received an intratumoral IFNγ injection after they received a vaccine containing 12 class I major histocompatibility complex-restricted melanoma peptides that increased vaccine-induced tumor-infiltrating lymphocytes. This cancer vaccine and tumor-directed IFNγ treatment enhanced T-cell infiltration and T-cell-mediated tumor control [27].

Granulocyte-macrophage colony-stimulating factor (GM-CSF) induces DC cell development and maturation as well as T-cell proliferation and activation to elicit innate and acquired immunity. A phase I clinical trial reported that intralesional injections of GM-CSF induced regression of subcutaneous metastases in melanoma patients with increased Langerhans cells and T-cell infiltration at injection sites, which correlated with clinical responses [28]. The combination of local treatment with different types of cytokines could be synergistic to stimulate multiple antitumor immunity. The combination of intratumoral IFNα-2b and IL-2 was tested in cystic glioblastoma, and the study found that dual cytokines could be safely injected into cystic glioblastoma without any evidence of side effects or an increase in surrounding tumoral edema, but 4 weeks of combined cytokine injection in ten glioblastoma patients had no effects on tumor progression [82]. Intratumoral injection of L19-IL2 and L19-TNF (both cytokines combining the human monoclonal antibody fragment L19) cytokine combination in patients with stage IIIC and IVM1a metastatic melanoma who were not candidates for surgery has shown that, in 20 efficacy-evaluable patients, 32 melanoma lesions achieved complete responses upon intralesional administration and complete responses in 7/13 (53.8%) noninjected lesions [29].

Most currently approved therapeutic Abs are antagonist that either directly bind to surface markers expressed on cancer cells like HER2, growth factors that support tumor growth like vascular endothelial growth factor, or regulatory proteins on cells like CTLA-4, PD-1 and PD-L1. Intratumoral injection of anti-CTLA4 antibodies in a slow-release formulation with an eight-fold lower dose of antibodies achieved tumor eradication as systemic delivery and resulted in thousand-fold decreased levels of antibodies in the serum, reducing adverse events and the risk of autoimmunity [83]. Intratumoral injection of anti-PD-1 plus ablative fractional laser in patients with basal cell carcinoma increased immune cell infiltration and reduced tumors including complete remission with limited side effects [31]. Intratumoral administration of anti-CTLA4 and intracavitary injection of anti-PD-1 combination tested in patients with resectable and unresectable recurrent glioblastoma (rGB) has resulted in an initial report that this combination is feasible and sufficiently safe to warrant further investigation in patients with rGB [30]. There are fewer clinical applications for agonist Abs because of the toxicity issues. For instance, the anti-CD28 monoclonal antibody that directly stimulates T cells, when tested in a phase 1 trial, with a single intravenous dose of the drug caused a severe cytokine storm response in six healthy young male volunteers [84]. To reduce such systemic toxicities, intratumoral delivery of agonistic Abs has been applied. In a mouse model humanized both for Fc receptors and CD40, direct delivery of an Fc-engineered anti-CD40 agonistic antibody to the tumor site maintained antitumor activity, but with fewer platelet and hepatic toxicities compared to systemic administration [85]. An initial clinical trial of intratumoral anti-CD40 antibodies in patients has reported that, for breast cancer with five patients, the best overall response was stable disease and, for melanoma with one patient in-transit disease, a complete response was observed in the second dose group and the toxicity was abrogated by intratumoral injection [32]. Another phase I trial of intratumorally injected agonistic CD40 antibodies has been conducted in patients with advanced solid tumors who had received standard of care treatments, and the results showed that this treatment was well tolerated at clinically relevant doses and associated with pharmacodynamic responses [33].

Antibody–drug conjugates (ADCs) are a new class of therapeutic Abs, which are composed of a monoclonal antibody that uses a chemical linker to conjugate a cytotoxic payload, allowing cytotoxic agents directed toward cells with a target antigen to be expressed on the cancer cell surface and bound by the Ab to reduce systemic exposure of cytotoxic agents [86]. The following three novel ADCs with different cytotoxic payloads have been recently approved: trastuzumab deruxtecan that targets HER2 for breast cancer, sacituzumab govitecan that targets Trop2 for breast cancer and enfortumab vedotin that targets Nectin4 for bladder cancer, all through systemic use [87]. Although ADCs have improved the overall survival in advanced cancer patients, the response rates and the outcomes of systemic ADCs are still unsatisfactory, and their systemic toxicities need to be addressed. For example, trastuzumab deruxtecan has been associated with a considerable risk of interstitial lung disease in a subset of patients [88]. To enhance the therapeutic potential of ADCs and to improve their therapeutic index, intratumoral injections of ADCs are being explored. With NCI-N87 tumor-bearing xenografts used as the animal model, the pharmacokinetic/pharmacodynamic behaviors of trastuzumab-vc-MMAE after intravenous, subcutaneous and intratumoral administrations were evaluated, and intratumoral administration was found to significantly increase tumor exposure and antitumor activity of the ADC with approximately a ~6-fold dose level reduction compared to the intravenous route [34].

### 2.3. Nucleic Acid-Based Gene Products

Due to advancements in genetic engineering technology, genes encoding cytokines can be inserted into DNA plasmids, mRNAs or viruses and locally injected into tumors, allowing cytokines to be durably expressed in situ compared to intratumoral injections of cytokine proteins. A phase II clinical trial was conducted in patients with stage III/IV melanoma by intratumorally delivering DNA plasmids encoding IL-12 (tavokinogene telseplasmid) into tumors using electroporation, and the results showed that the treatment was well tolerated and led to systemic immune responses, tumor regression and increased immune infiltration even in untreated/distal lesions, but adaptive immune resistance limited the response [35]. Intratumoral electroporation of tavokinogene telseplasmid with PD-1 blockade increased immune cell infiltration and supported the IL-12/IFNγ feed-forward cycle, driving an intratumoral cross-presenting dendritic cell subpopulation with increased tumor infiltrating lymphocytes (TILs) and induced a higher than expected response rate in the patients with advanced melanoma, with a low frequency of checkpoint-positive cytotoxic lymphocytes [36].

When transfected into cells, synthetic mRNAs can be used as templates for the synthesis of designed proteins and peptides, whose properties make mRNAs encoding gene products a novel therapeutic modality. With nucleoside modification and elimination of double-stranded RNA plus the use of nanoparticle-based formulations, the immunomodulatory activity of mRNAs has been reduced and the transfection efficiency of mRNAs has been increased, thus resulting in increased and prolonged expression of designed products such as cytokines, costimulatory receptors or therapeutic antibodies [89]. In preclinical models, a single intratumoral dose of mouse (m)IL12 mRNA stimulated IFNγ and CD8+ T-cell-dependent tumor regression in multiple syngeneic mouse models, and mice with a complete response showed memory immunity to rechallenge, in which antitumor activity was not linked to NK and NKT cells and was enhanced by anti-PD-L1 [90]. Intratumoral injection of MEDI1191 (IL-12 mRNA), a lipid nanoparticle (LNP)-formulated therapy designed to generate local IL-12 production and induce anenestic antitumor activity, has been tested in a phase I study in sequential or concurrent combination with durvalumab, an anti-PD-L1 antibody, in patients with advanced solid tumors with superficial and deep-seated lesions. The results reported that the sequential combination of intratumoral MEDI119 with systemic durvalumab showed a safe profile, encouraging preliminary antitumor and pharmacodynamic activity [37]. Direct intratumoral delivery of lipid nanoparticle encapsulating messenger RNAs (mRNA-2752) encoding cytokines IL-23, IL-36γ and T-cell co-stimulator OX40L increased complete response rates in treated and untreated distal tumors compared to the cytokine mRNAs only treatment in mouse models and animals, and complete responses were subsequently protected from tumor rechallenge due to multiple downstream cytokine expression. Immune cell recruitment into tumors and the combination of triplet mRNA and checkpoint blockade have led to efficacy in systemic immune checkpoint resistant tumor models [91]. This intratumoral mRNA-based therapy has been in clinical development alone and in combination with durvalumab for treating patients with solid tumors, and preliminary evidence of acceptable tolerability and tumor response as well as increased proinflammatory cytokines (e.g., IFN-γ and TNF-α) and PD-L1 expression predominantly in tumor-associated immune cells have been obtained [38]. Local intratumoral administration of SAR441000, a mixture of four mRNAs encoding IL12 single chain, IFNα2b, GM-CSF and IL15sushi, in immunocompetent mice, has shown successful antitumor immunity both in injected and noninjected tumors; in addition, combining the mRNAs with checkpoint inhibitors further enhanced antitumor responses, leading to tumor eradication and prolonged survival. Thus, a clinical study was initiated to investigate the effect of intratumoral administration of SAR441000 monotherapy and in combination with cemiplimab (an anti-PD-1 Ab) in patients with advanced solid tumors [39]. Intratumoral delivery of mRNA encoding the costimulatory molecule CD70, the CD40 ligand and constitutively active Toll-like receptor 4 (TriMix mRNA) has shown systemic therapeutic antitumor immunity in various mouse cancer models through modulating the activity of tumor-infiltrating dendritic cells (TiDCs) [40]. Circular mRNA (cmRNA) has the capacity to generate a more potent and durable protein expression, but requires a simpler manufacturing procedure. Direct intratumoral injection of cmRNA encoding a mixture of cytokines has been shown to enhance intratumoral and systematic antitumor immune responses and to increase anti-PD-1 antibody-induced tumor suppression in a syngeneic mouse model, suggesting the potential of the cmRNA platform in intratumoral immunotherapy [41].

### 2.4. Microbes

Spontaneous tumor regression has been recorded after viral or bacterial infections, which has provided the basis for clinical trials to use viruses or bacteria as therapeutic agents to treat cancer patients. Selected and genetically modified viruses with the capability to specifically infect and propagate in tumor cells have been used to destroy tumor cells and to stimulate the innate immune response as well as adaptive antitumor immunity to eradicate distant uninfected tumor cells, called oncolytic virotherapy [92,93].

#### 2.4.1. Viruses

Talimogene laherparepvec (T-VEC) is a herpes simplex virus 1, genetically modified to express GM-CSF and selectively infect cancer cells. So far, T-VEC is the only approved viral cancer immunotherapy for local treatment of unresectable cutaneous, subcutaneous, and nodal lesions in patients with melanoma recurrent after initial surgery [94]. In the phase III OPTiM study, which supported the approval of T-VEC, patients with unresectable stage IIIB-IVM1c melanoma who were given an intratumoral injection of T-VEC were found to have a longer survival, 5-fold increase in objective response rate and 24-fold in complete response rate as compared with those who received subcutaneous GM-CSF, and the treatment was well tolerated [42]. In a phase II trial, patients with stage 2/3 TNBC, treated with intratumoral T-VEC injections and neoadjuvant chemotherapy, i.e., paclitaxel followed by doxorubicin and cyclophosphamide regimens, and then surgery, were assessed, and the results revealed that immune activation during treatment had a correlative relationship with response [95]. To expand its application, currently, intratumoral T-VEC is being further investigated in a phase I/II trial, combining intravenous pembrolizumab in patients with hepatocellular carcinoma (HCC) or liver metastases [96] or in a phase II study for patients with inoperable locoregional recurrence of breast cancer [43]. Furthermore, T-VEC has also been tested in combination with ipilimumab (an anti-CTLA-4 Ab) versus ipilimumab alone in patients with advanced melanoma in a phase II study; the trial results were significant with a higher objective response rate of 39% in the combination group as compared with 18% in the ipilimumab group, and responses were not restricted to injected lesions with visceral lesion decreases observed in 52% of patients in the combined treatment group and 23% of patients in the ipilimumab-treated group with no additional safety concerns versus ipilimumab [44]. T-VEC has also been combined with an anti-PD-1 antibody in the phase III, randomized, double-blind MASTERKEY-265/KEYNOTE-034 study in patients with unresectable stage IIIB-IVM1c melanoma who were naive to anti-PD-1 therapy with injectable lesions; the results revealed that T-VEC in combination with intravenous pembrolizumab did not significantly improve PFS or OS vs. a placebo plus pembrolizumab, and no new safety concerns were identified [97]. So far, these explored indications for T-VEC have not been FDA-approved.

Other oncolytic viruses are also being studied in preclinical studies and clinical trials to identify more intratumoral virotherapy options. CAVATAK, an oncolytic immunotherapy, is a bioselected oncolytic strain of coxsackievirus A21 (CVA21) that preferentially infects intercellular adhesion molecule 1 expressing tumor cells. Intratumoral injection of CAVATAK to patients with advanced melanoma, in the phase II CALM study, increased immune cell infiltration and immune-related response genes in tumors [45]. Canerpaturev (C-REV), originally isolated from the herpes simplex virus 1 (HSV-1) strain HF as clone 10 (also known as HF10), has a unique dsDNA genomic structure with non-engineered, two deletions resulting in attenuated pathogenicity and neuroinvasiveness [98]. Intratumoral administration of the herpes simplex virus HF10 has been studied in patients with recurrent head and neck squamous cell carcinoma and it has been shown that HF10 replicated, distributed well in the tumor nodules and resulted in cell death in a major population of tumor cells, with no significant adverse effects [99]. In a pilot study, HF10 was intratumorally injected into cutaneous or subcutaneous metastases of breast cancer patients, and the study found that the treatment showed tolerability with no adverse effects and caused from 30% to 100% cancer cell death in metastatic nodules [46].

Intratumoral HF10 plus ipilimumab combination treatment tested in a phase II trial in stage IIIB, IIIC or IV unresectable ipilimumab naïve melanomas has demonstrated a favorable benefit/risk profile and encouraging antitumor activity in patients with stage IIIB, IIIC or IV unresectable or metastatic melanoma [47]. Direct administration of HF10 using endoscopic ultrasound (EUS)-guidance into unresectable locally advanced pancreatic cancer in combination with erlotinib and gemcitabine treatment has been studied, and it has been shown that this virotherapy, targeted and chemotherapy combination, was a safe treatment with antitumor signals [48].

Pexa-Vec (pexastimogene devacirepvec, JX-594) is an oncolytic vaccinia virus with the thymidine kinase gene inactivated and genetically modified to express human GM-CSF for selective replication in cancer cells, with cell-cycle defects and epidermal growth factor receptor/Ras pathway activation to cause direct oncolysis once infecting cancer cells, plus its GM-CSF expression that also stimulates shutdown of tumor vasculature and antitumor immunity [100]. Intratumoral injection of Pexa-Vec in patients with refractory primary or metastatic liver cancer, in general, has been well tolerated and has exhibited a dose-related survival benefit [101]. However, the interim futility analysis of the phase III PHOCUS trial of intratumoral Pexa-Vec plus sorafenib (a multitargeted tyrosine kinase inhibitor, which is the standard of care first-line systemic treatment for advanced HCC) versus sorafenib alone in patients with advanced HCC without prior systemic therapy has indicated that the trial is unlikely to meet its primary overall survival objective by the time of the final analysis [49,50]. In addition, it has been examined whether Pexa-Vec plus best supportive care improved overall survival compared to best supportive care alone in patients with HCC who failed sorafenib therapy, and again, despite a tolerable safety profile and induction of T-cell responses, it failed to improve survival as a second-line therapy after sorafenib failure [51]. Teserpaturev/G47∆ is a triple-mutated, third-generation oncolytic herpes simplex virus type 1 (HSV-1) and currently, it has been approved for malignant glioma treatment in 2021 in Japan. The approval of teserpaturev/G47∆ was supported by a single arm clinical phase II study in patients with residual or recurrent, supratentorial glioblastoma, after receiving radiation treatment and temozolomide, in which the stereotactic administration of teserpaturev/G47∆ intratumorally demonstrated a 1-year survival rate of 84.2% with an increasing number of tumor-infiltrating CD4+/CD8+ T cells and persistently low numbers of Foxp3+ cells in biopsies [52].

Tasadenoturev (DNX-2401) is an oncolytic replication-competent adenovirus with a 24 bp deletion in the E1A region of the genome, enabling the virus to replicate in cancer cells selectively and efficiently. In a preclinical study of a mouse GL261-glioma model, intratumoral DNX-2401 induced cytotoxic effects in mouse glioma cells. Viral treatment in GL261-glioma-bearing mice increased the infiltration of innate and adaptive immune cells into tumors, stimulating Th1 immunity at the tumor site, which elicited specific anti-glioma immunity [102]. Additionally, DNX-2401 has been tested in a phase I trial in 37 patients with malignant glioma, with a single intratumoral administration of DNX-2401 into biopsy-confirmed recurrent tumors; the results showed that 20% of the patients survived more than 3 years from treatment, and among them, three patients had a ≥95% reduction in the enhancing tumor (12%), with all three of these significant responses resulting in more than 3 years of progression-free survival since the treatment started [53]. DNX-2401 was shown to replicate and spread within the tumor, and tumor infiltration by CD8+ and T-bet+ cells as well as transmembrane immunoglobulin mucin-3 downregulation after treatment were observed [53]. To maximize antitumor immune responses, the combination of intratumoral injection of oncolytic virus DNX-2401 followed by intravenous anti-PD-1 antibody pembrolizumab was evaluated in a phase I/II trial in patients with recurrent glioblastoma; whereas the primary efficacy endpoint objective response rate was not met, the combined treatment was safe and improved survival as well as clinical benefits were observed [54]. ONCOS-102 (AdV5/3-Δ24-GM-CSF), which is a serotype 5 adenovirus, with a chimeric capsid for enhanced gene delivery and a 24 bp deletion in the Rb binding site of the E1A region to restrict cancer cell replication and engineered GM-CSF expression for an enhanced immunostimulatory effect, is another adenovirus that has been studied in clinical trials. In a phase I trial, 12 patients with refractory solid tumors were repeatedly treated with ONCOS-102 intratumorally in addition to daily low-dose oral cyclophosphamide; this treatment caused increased immune cell infiltration into the tumors and stimulated TH1 cells and TH1 type immune gene profile as well as disease control for 40% of the patients [55]. The upregulation of PD-L1 expression in tumors in a subset of patients with mesothelioma was observed, which supported the following pilot study to investigate ONCOS-102 plus anti–PD-1 therapy in anti-PD-1-resistant melanoma. In this study, ONCOS-102 in addition to pembrolizumab were well tolerated and caused persistent immune-related gene expression and T-cell infiltration as well as tumor reduction even in noninjected lesions [103].

Reolysin (pelareorep) is a human reovirus type 3 strain with direct oncolytic activity in Ras-activated tumor cells and the ability to elicit dendritic cell maturation as well as NK cell activation and recruitment. The combination of local intratumorally delivered reovirus with systemic immune checkpoint inhibition in a murine subcutaneous B16 melanoma model increased survival compared to untreated and monotherapy groups by increasing NK killing of virus-infected cells and inhibited immune suppression caused by Foxp3+ Treg cells [104]. A phase II trial was conducted in treatment-naive metastatic pancreatic adenocarcinoma patients who received either paclitaxel/carboplatin plus pelareorep or paclitaxel/carboplatin, and the results showed there was no difference in progression-free survival (PFS) between these two treatments [56]. Coxsackievirus A21 (CVA21, Cavatak) is a non-enveloped virus with a single-stranded RNA genome with preferential infection of intercellular adhesion molecule 1 (ICAM-1)-expressing cells. A phase II study of intratumoral CVA21 injection in treated or untreated unresectable stage IIIC-IVM1c melanoma patients met its primary endpoint with 21 of 57 (38.6%) evaluable patients displaying immune-related PFS at 6 months, with a median irPFS of 4.2 months [45]. Recombinant nonpathogenic polio-rhinovirus chimera (PVSRIPO) is a genetically engineered polio virus that selectively targets glioma cells expressing Necl-5 to minimize neurovirulence. Intratumoral infusion of PVSRIPO in patients with recurrent WHO grade IV malignant glioma showed overall survival among the patients who received PVSRIPO, which reached a plateau of 21% at 24 months that was sustained at 36 months [57].

#### 2.4.2. Bacteria

The use of bacteria to target tumors has been explored as a unique therapeutic option since Dr. Coley’s efforts to solve the ongoing challenges of cancer treatment. However, until now, only Bacillus Calmette–Guérin (BCG), a strain of Mycobacterium bovi, has been approved only for the intravesical treatment of non-muscle invasive bladder cancer (NMIBC) to prevent recurrence [58], although, decades ago, intratumoral BCG demonstrated complete remission of transplantable hepatomas in guinea pigs [105]. Other malignancies such as lung cancer and melanoma have been tested in clinical trials, using intralesional injection of BCG into tumors as a therapeutic approach, but no more indication of BCG has been approved. However, the application of bacteria and bacterial-based products as treatments to combat cancer are still being explored.

*Salmonella typhimurium* is a promising cancer immunotherapy option because it can be modified and proliferates both in non-hypoxic and hypoxic tumor sites. Intratumoral delivery of recombinant attenuated Salmonella enterica serovar typhimurium vaccine significantly repressed Her-2/neu-expressing tumor growth by causing transformation of immunosuppressive MDSCs into TNF-α-secreting neutrophils and decreasing the generation of Treg cells, especially in the presence of tumor-specific CTLs [106]. Intratumoral injection of an attenuated strain of *Salmonella typhimurium*, designated VNP20009, generated by deletion of the msbB and purL genes, was tested in a phase I trial and showed a safe profile [59]. Intratumoral injection of *S. typhimurium* induced tumor cell apoptosis, decreased tumor angiogenesis and inhibited the growth of the injected schwannoma tumors in two murine schwannoma models; adding anti-PD-1 antibodies to the intratumoral injection of *S. typhimurium* had an additive effect on suppressing schwannoma growth [107]. Another promising bacterial vector for cancer immunotherapy is Listeria monocytogenes, a Gram-positive, facultative anaerobic bacterium, which can be engineered to express proteins such as tumor-associated or specific antigens to enhance specific antitumor immunity. There have been a number of clinical trials conducted to test the intravenous route of Listeria monocytogene-based vaccines on different cancers such as cervical, lung, pancreatic and prostate cancers [108]. One injection of high dose Listeria monocytogenes in a tumor area, followed by 14 intraperitoneal injections of a lower dose treatment, was tested in a genetically engineered mouse model of metastatic melanoma and this approach decreased not only primary tumors but also metastatic nodules with increased cytokine expression and T-cell infiltration in tumors [109].

*Clostridium novyi* is a highly mobile bacterium and a spore-forming organism that is extremely sensitive to oxygen. Intratumoral injection of *Clostridium novyi* (*C. novyi*-NT, non-toxic) spores, an attenuated strain of *Clostridium novyi*, has been performed in companion dogs bearing spontaneous solid tumors with an objective response rate of 37.5% (6 of 16 dogs, three complete and three partial responses) [110]. This canine field study led to the testing of a human patient who had an advanced leiomyosarcoma with an intratumoral injection of *C. novyi*-NT spores, and this treatment reduced the tumor within and surrounding the bone [110]. A first-in-human study that enrolled patients with treatment-refractory solid tumors who received a single intratumoral injection of *C. novyi*-NT showed that among 22 evaluable patients, 9 patients (41%) had a decrease in the size of the injected tumor and 19 patients (86%) had stable disease as the best overall response in injected and noninjected lesions combined [60]. A further clinical study for intratumoral administration of *C. novyi*-NT to treat patients with injectable, treatment-refractory solid tumors in combination with intravenous pembrolizumab demonstrated a manageable toxicity profile of intratumoral *C. novyi*-NT plus systemic pembrolizumab, which resulted in partial responses in two of nine patients (tongue squamous cell cancer and nasopharyngeal cancer) [111].

### 2.5. Cells

Human immune cells including DC and T cells can be isolated from patients (autologous) or donors (allogeneic) for ex vivo expansion and further modification with stimulating factors or genetic engineering to produce clinical-grade cell therapies. For example, Sipuleucel-T, which has been approved for prostate cancer, consists of autologous peripheral-blood mononuclear cells, including APCs, that have been stimulated ex vivo with a recombinant fusion protein (PA2024), which is a prostate antigen, prostatic acid phosphatase fused to GM-CSF, an immune cell activator [112]. The other example is the first approved CAR-T for B-cell leukemia, tisagenlecleucel, produced ex vivo with the use of autologous T cells transduced with a genetically modified lentiviral vector to express a CAR-targeting CD19 and a CD3-zeta domain to produce a T-cell activation signal and a 4-1BB (CD137) domain to generate a costimulatory signal [113]. The application of these modified immune cells is enormous, but most of them have been applied systemically through intravenous administration. To circumvent the disadvantages of systemic immune cell therapy, the approach of injecting immune cells intratumorally has been pursued in preclinical and clinical studies. Moreover, to prevent the tedious and uncertain tasks associated with neoantigen identification and characterization as well as in vitro production of tumor neoantigens, patients’ existing tumors (primary or distant) have been used as direct neoantigen sources. By injecting DCs directly into a patient’s own tumor, it would allow the vaccine to directly enter into the patient themselves, thereby minimizing the resource allocation required in ex vivo processing. Additionally, this strategy makes the best use of the complete neoantigen repertoire in a patient’s own tumor rather than being limited to a defined number of identified and characterized ex vivo generated tumor neoantigens. The safety and efficacy of intratumoral injection of DCs, generated from monocytes obtained by phlebotomy with GM-CSF and IL-4 in autologous plasma into the metastatic dermal or subcutaneous tumors of patients with melanoma and breast carcinoma, were examined in a pilot clinical study. Regression of the injected tumors was observed in some patients with increased lymphocyte infiltration [61]. Intratumoral administration of proinflammatory allogeneic DCs induced antitumor immune responses and prolonged survival in patients with unfavorable risk metastatic renal cell carcinoma (mRCC) with standard tyrosine kinase inhibitors (TKI), in a randomized, multicenter, phase II mRCC trial [114]. Using the experimental mouse models of renal cell cancer and MethA sarcoma, intratumoral administration of DCs, genetically modified to express IL-12, IL-21 or IFNα, demonstrated potent therapeutic effects against established tumors with the induction of potent tumor antigen-specific CD8+ T-cell responses and increased CD4+ and CD8+ T cell infiltration in tumors [115]. Ilixadencel, made of allogeneic inflammatory DCs, can be used as a cell-based immune primer by injecting them intratumorally. The safety and efficacy of ilixadencel in patients with progressing advanced/metastatic gastrointestinal stromal tumors despite ongoing treatment with second or later lines of TKIs were assessed in a phase I clinical trial; intratumoral ilixadencel treatment had an acceptable safety profile and tumor responses were detected in 33% of treated patients [116]. To evaluate the clinical effects of intratumoral ilixadencel on mRCC in combination with nephrectomy and sunitinib, compared with nephrectomy and sunitinib monotherapy, a randomized phase 2 study was conducted; the results found that the study failed to meet its primary endpoints, but the ilixadencel and sunitinib combined treatment was correlated with a numerically higher, nonsignificant, confirmed response rate, including complete responses as compared with sunitinib treatment alone [117]. To re-boost antitumor T-cell immunity in tumors, adding intratumoral allogeneic dendritic cells as an immune-priming step, with anti-CTLA-4 Abs, could improve efficacy. Using an established CT-26 tumor model, AlloDCs and anti-CTLA-4 Abs combined treatment significantly enhanced the effectiveness, with 70% of mice being cured and enhanced infiltration of activated antigen-presenting endogenous DCs and CD8+ T cells with a tissue-resident memory phenotype (CD49a+CD103+) observed [62].

The adoptive T-cell therapy using TILs or CAR-T cells has demonstrated clinical benefits for patients with melanoma (TILs) and patients with hematological malignancies (CAR-T cells) through the systemic intravenous infusion of ex vivo expanded TILs or genetically modified T cells, however, with the challenges of T-cell exhaustion and an immunosuppressive tumor microenvironment [118]. Intratumoral T-cell therapy could be an option to solve this problem. In a mouse liver tumor model, intratumoral injection with activated TILs and simultaneous administration of recombinant interleukin 2 (rIL-2) and IFNα was more effective in regressing the mouse liver tumor than the IL-2 + IFNα combination [119]. A case reported that intratumoral adoptive immunotherapy with TILs in patients with melanoma, in combination with subcutaneous infusion of IL-2 and a single intratumoral injection of a low dose of IFNα, led to consistent tumor regression [63]. With the enormous clinical successes of CAR-T cells in B-cell leukemias and lymphomas, but not for solid tumors with the intravenous route, direct injection of CAR-T cells with different genetic modifications into tumors could improve the efficacy in solid malignancies for this class of therapies [64]. Repeated intratumoral injections of CAR-modified T cells specific to erbB-2 accumulated cells at tumor sites have eliminated tumor cells as well as prevented relapse in a mouse spontaneous mammary tumor model due to overexpression of the human erbB-2 transgene [120]. A phase I dose-escalation study was conducted for intratumoral CAR-T cell therapy, in which intratumoral ErbB-targeted T4 CAR-T cells were tested in patients with squamous cell carcinoma of the head and neck following locoregional recurrence, and the safety of intratumoral injection of T4 CAR-T immunotherapy was shown [64]. In a phase 0 clinical trial, intratumoral administration of mRNA-transfected c-Met-CAR-T cells was applied on patients with metastatic breast cancer with accessible cutaneous or lymph node metastases; it was reported that mRNA c-Met-CAR T cell injections were well tolerated and caused tumor necrosis which was surrounded by macrophages [65].

Xenogeneic cell (e.g., pig cells) therapy has been proposed to treat tissue failures and degenerative diseases such as type I diabetes and Parkinson’s disease through cell replacement to restore the function of tissue failures; however, so far, there is no approval for xenogeneic cell therapy, because the body’s immune responses to xenogeneic cells are powerful and multifaceted, involving innate immune components and adaptive immunity of T cells and antibodies to reject the cells [121]. However, compared to antitumor immunity with xenogeneic cell rejection by the body’s immune system, both share similarities in triggering the body’s innate and adaptive immunity. The immune system can recognize both its own and non-self cells due to tumor neoantigens and tumor-associated carbohydrates/lipids in tumor cells and xenoantigens and xeno-carbohydrates/lipids in xenogeneic cells [122]. Thus, we have proposed to use xenogeneic tissue cells as an intratumoral therapeutic agent to treat cancers of the same histologic types, in which tumor cells express orthologous proteins as their xenogeneic tissue cell counterparts, so when xenogeneic tissue cells are injected into tumors, various immune reactions could be stimulated and turn into antitumor immunity similar to that of the xenogeneic DNA vaccine [123]. Recently, our group demonstrated that intratumoral injection of xenogeneic urothelial cells alone suppressed tumor growth and the efficacy was enhanced when combining chemotherapy both on injected and noninjected tumors in two murine syngeneic models of bladder cancer. Intratumoral immune cell infiltration and systemic activation of immune cell cytotoxic activity, cytokine IFNγ production and proliferation ability as well as elevated chemokine CXCL9/10/11 levels in tumors were observed in mice receiving intratumoral therapy. These data suggest that intratumoral xenogeneic urothelial therapy could be applied in the treatment of advanced bladder cancer as a locoregional therapy that intratumorally administers xenogeneic cells into either primary or distant tumors. This new therapeutic option, eliciting both local and systemic antitumor immunity, would complete the comprehensive cancer management along with systemic therapeutic modalities [67]. Similar results have been found with intratumoral xenogeneic mammary cell therapy in a mouse breast tumor model and intratumoral xenogeneic pancreatic cell treatment in a mouse pancreatic tumor model, turning immunologically “cold” tumors into “hot” tumors [66,124]. By injecting highly immunogenic xenogeneic tissue cells into tumors with low immunogenicity or developed tolerance, xenoantigens, xeno-carbohydrates and lipids could initiate immune responses in situ to target non-self cells, including tumor cells, breaking their tolerance. Our findings demonstrate that xeno rejection from the body toward xenogeneic cells could be utilized to treat cancers in a similar way as Dr. Coley used bacteria to treat cancers because the body’s antibacterial immunity turns on bystander antitumor immunity, adding a new class of intratumoral therapeutic agents into the cancer immunotherapy armamentaria.

## 3. Clinical Challenges for Intratumoral Immunotherapy with Immune Modulators

Oral, intramuscular and intravenous medication administration routes have been applied routinely; however, in clinics, intratumoral injection is a new way to deliver drugs to the body. Currently, there is only one FDA-approved indication with the intratumoral approach for patients with melanoma; others are still in clinical trials and preclinical studies. There are several gaps and/or needs that require more studies on intratumoral immunotherapeutic agents. Several key issues need to be resolved before intratumoral immunotherapy could fulfill its promise.

### 3.1. Feasibility of Intratumoral Injection Tumor Size and Location Limits

Intratumoral injection has not been used in routine clinical practice. The safety of this emerging procedure is a prime concern in identifying lesions that are safely accessible and injected without causing tumor spread and hemorrhage. As mentioned previously, T-VEC, the first and only FDA-approved oncolytic viral therapy for the local treatment of unresectable cutaneous, subcutaneous, and nodal lesions in patients with melanoma recurrent after initial surgery, has been designed to be injected into nodal tumor lesions that are visible, palpable or detectable by ultrasound guidance [94]. Thus, it has been clinically validated that tumors located in visible cutaneous, subcutaneous or cervical mucosal sites or palpable lymph nodes could be injected with or without ultrasound guidance. For visceral tumor sites in lung, liver, and GI or GU tracts, imaging (ultrasound, computed tomography and magnetic resonance) or endoscopic guidance is required for intratumoral injection, which has been tested in clinical trials, for example, intratumoral T-VEC injection with ultrasound/computed tomography guidance into liver tumors and non-HCC liver metastases (NCT02509507). The advent of image-guided procedures has allowed increased accessibility of tumors across a range of histological conditions and target organs, which has made intratumoral administration of immunotherapeutic agents more feasible [125]. There are tumor lesions that are accessed via surgical procedures, for example, intratumoral infusion of the carcinoembryonic antigen-expressing measles virus through a stereotactically placed catheter within the tumor (NCT00390299) and endoscopic ultrasound-guided fine-needle injection has been adopted to deliver allogeneic mixed lymphocyte culture into tumors of patients with advanced pancreatic carcinoma [126]. Consequently, these local injections into tumor sites by inserting a delivery device with or without imaging or endoscopic guidance could be applied in standard of care, and a number of clinic trial results could define the safety and plausibility of this administration procedure. A risk assessment needs to be performed before administration to minimize complications (e.g., hemorrhage) by considering tumor texture, size and location as well as the consequence of injection. For example, in the case of T-VEC [94], the size (longest dimension) of injected tumors could be ≤0.5 cm and >5 cm, but from the largest to the smallest lesion, and all injectable sites would be injected until the maximum cumulative dose of 4 mL per visit is reached. The total injection volume for each treatment visit should not exceed 4 mL for all injected lesions combined. A lesion size >5 cm is injected up to 4 mL, a lesion from >2.5 cm to 5 cm is injected up to 2 mL, a lesion size from >1.5 cm to 2.5 cm is injected up to 1 mL, a lesion size from >0.5 cm to 1.5 cm is injected up to 0.5 mL and a lesion size ≤0.5 cm is injected up to 0.1 mL. T-VEC can be injected evenly and completely within a lesion by pulling the needle back without exiting the lesion, with redirection of the needle as many times as necessary while injecting the remainder of the dose to ensure the full dose is dispersed evenly and completely. Other intratumoral injection procedures could also be standardized with the help of imaging or endoscopic guidance to allow homogeneous distribution of immunotherapeutic agents without the risk of leakage.

The ideal intratumoral immunotherapy regimen (dosage and timing of injection) for each immunostimulatory agent that gives the optimal antitumor response has to be determined depending on their mechanisms of action and local PK/PD properties. The injection volume could be determined by the tumor size, but the drug amount (weight for small and macromolecules, colony forming unit for bacteria, plaque forming unit for viruses and cell number for cell products) would vary among different therapeutic classes, similar to the treatment schedule. For T-VEC, the starting initial dose is up to a maximum of 4 mL at a concentration of 10^6^ plaque-forming units (PFU) per mL and, after 3 weeks, second and subsequent doses are administered up to 4 mL at a concentration of 10^8^ PFU per mL every two weeks for at least 6 months, unless other treatment is required or until there are no injectable lesions to treat [94]. In a clinical trial, image-guided intratumoral injection of JX-594 with 10^9^ or 10^8^ PFU using a multi-pronged Quadrafuse needle was administered to ensure even distribution of the virus throughout the tumor when possible, into up to five intrahepatic tumors at different time points, i.e., days 1, 15 and 29, with the volume of JX-594 solution to be injected proportionally to the volume of the tumor to be injected (25% of the tumor volume) [101]. The regimen for each agent needs to be determined based on the results of pharmacology, toxicology and clinical trials.

### 3.2. Intratumoral Immunotherapy Pharmacology and Toxicology

In conventional pharmacology for oral or intravenous routes, there are four pharmacokinetic characteristics of a drug that need to be effectively determined, i.e., absorption, distribution, metabolism and excretion (ADME), which describe how a drug moves and transforms in the body, causing various drug level exposures within tissues. However, intratumoral administration directly injects the drug into the effect sites, where a high initial tissue concentration occurs, and then the drug diffuses throughout the injected sites and into the systemic circulation over time. The pharmacokinetics of locally injected agents are not easily defined as systemic administration. The pharmacokinetic framework for intratumorally injected agents would focus on local tissue/tumor exposure and retention to correlate with therapeutic efficacy and exert maximal therapeutic effect [127]. Due to the variations in different classes (size and chemical/physical properties) and the biological reactions to the different agents in the body, the pharmacokinetic profiles would be very different from one agent to another. For living drugs, such as viruses, bacteria and cells, the biodistribution and clearance, shedding and replication should be addressed.

The identification of biomarkers to correlate local activity with systemic efficacy is essential in intratumoral immunotherapy pharmacodynamic studies, which could include cytokines, antibodies and immune cells [14]. The immunotoxicity, i.e., the unintended (toxicological) actions on the immune system, of immune-modulating drugs affects the safety of the agents. Toxicology studies of each drug need to be performed in preclinical and clinical studies to identify irAEs to guide clinical safe dosing [128]. A dose-escalation clinical trial of intratumoral immunotherapy must be designed to have clear, dose-related dose-limiting toxicities to identify the maximum tolerated dose, and then the recommended phase 2 dose.

### 3.3. Clinical Outcome Assessment and Application

To measure the outcome of intratumoral immunotherapy, the objective response rate of tumor responses has been measured by radiological assessment of injected and noninjected sites using the intratumoral immunotherapy-specific Response Criteria for Intratumoral Immunotherapy in Solid Tumors (itRECIST), which is based on regression of both injected and noninjected lesions [129]. By applying itRECIST to both injected and noninjected tumor lesions, both responses are reported separately. Other endpoints like disease control rate, duration of response, and the survival of responders are used to assess clinical efficacy. In cancer clinical research, the trials’ test experimental therapies with unproven safety and effect in humans are studied first in patients with terminal advanced-stage malignancies and, if proven safe and efficacious in advanced cancers, then the treatments are further studied for their safety and effects in early stage cancers [130].

## 4. Conclusions

Intratumoral immunotherapy provides several advantages over conventional systemic cancer immunotherapy strategies by maximizing the therapeutic index values of different immunotherapeutic agents and reducing systemic exposure. In addition, as a local treatment and targeting the body’s immune system therapy, intratumoral immunotherapy offers a therapeutic option to combine with systemic chemo- and targeted therapies as well as regional surgery and radiotherapy to form a comprehensive cancer treatment. With all the therapeutic options (systemic vs. locoregional and tumor targeting vs. body targeting) available to patients as well as the advancement of precision medicine to find targets and immunotherapy to strengthen the body’s immune system, it allows a combination treatment plan that selects these treatment options to comprehensively manage cancer patients. Complete cancer management that follows the dogma of Eastern medicine, i.e., “to cure the diseases and let the body return to a healthy state, medicines need to treat the causes of diseases and treat the body that responds to the diseases” would transform cancer management to bring best outcomes to patients.

## Figures and Tables

**Table 1:** Current development of immunotherapeutic agents used for intratumoral immunotherapy.

Class	Agent	Target Tumor Type	Development Stage	Main Result	Reference
Small and macromolecule	Imiquimod	Mesothelioma	Preclinical murine model	30% Complete resolution	[17]
	MIW815 (ADU-S100)	Solid tumors or lymphomas	Phase Ib	10.4% Overall response rate	[18]
	G100	Lymphoma	Phase I/II	33.3% Overall response rate	[19]
	CpG oligodeoxynucleotide	Colon; Breast	Preclinical murine model	Survival benefit	[20]
	Vidutolimod	Lung	Phase Ib	15.4% to 25.0% Response rate	[21]
	α-gal glycolipid	Advanced solid tumors	Phase I	No DLT	[22]
Peptides and Proteins	LTX-315 (ruxotemitide)	Advanced solid tumors	Phase I	Immune-mediated anticancer activity	[23]
	IL-2	Melanoma	Phase II	36.7% Overall response rate	[24]
	IFNα-2a	Basal cell carcinoma	Clinical study	55% Complete remission	[25]
	IFNα-2b	Basal cell carcinoma	Clinical study	80% Cured	[26]
	IFNγ	Melanoma	Clinical study	Enhance T-cell infiltration and mediated tumor control	[27]
	GM-CSF	Melanoma	Phase I	23% Partial regression	[28]
	L19-IL2 and L19-TNF	Melanoma	Phase II	20 Efficacy-evaluable patients, 32 melanoma lesions complete responses	[29]
	Anti-CTLA4	Glioblastoma	Phase I	34% Two-year overall survival rate	[30]
	Anti-PD-1	Basal cell carcinoma	Phase I	45% Tumor reduction ≥25%	[31]
	Anti-CD40	Breast; melanoma; advanced solid tumors	Phase I	Clinical activity observed	[32,33]
	Trastuzumab-vc-MMAE	Gastric carcinoma	Preclinical murine model	Increased antitumor activity	[34]
Nucleic acid-based gene products	tavokinogene telseplasmid	Melanoma	Phase II	35.7–41% Overall response rate	[35,36]
	MEDI1191	Advanced solid tumors	Phase I	Preliminary antitumor activity	[37]
	mRNA-2752	Advanced solid tumors	Phase I	5.8% Overall response rate	[38]
	SAR441000	Advanced solid tumors	Phase I	Generally, well tolerated	[39]
	TriMix mRNA	Lymphoma; mastocytoma; lung	Preclinical murine model	Delay the growth of established tumors	[40]
	Circular mRNA	Lung; melanoma; colon	Preclinical murine model	Tumor repression	[41]
Viruses	Talimogene laherparepvec	Melanoma (only FDA-approved indication)	Phase III	31.5% Overall response rate	[42]
	Talimogene laherparepvec	Breast; liver	Phase II	31.5–52% Overall response rate	[43,44]
	CAVATAK	Melanoma	Phase II	28.1% Overall response rate	[45]
	Hf10	Breast; melanoma; pancreatic;	Phase I; phase II	25–41% Overall response rate	[46,47,48]
	Pexastimogene devacirepvec	Liver	Phase III	Fail to improve survival	[49,50,51]
	Teserpaturev	Glioblastoma	Phase II	84.2% One-year survival rate	[52]
	Tasadenoturev	Glioma	Phase I	20% Patients survived >3 years	[53,54]
	ONCOS-102	Refractory solid tumors	Phase I	40% Disease control	[55]
	Reolysin	Pancreatic	Phase I	No difference in progression-free survival to chemotherapy	[56]
	Cavatak	Melanoma	Phase II	38.6% Progression-free survival at 6 months	[56]
	PVSRIPO	Glioma	Phase 1	21% Survival at 24 months	[57]
Bacteria	Bacillus Calmette–Guérin	Non-muscle invasive bladder cancer (only FDA-approved indication)	Phase II	76% Complete remission	[58]
	VNP20009	Advanced or metastatic cancer	Phase I	Report a safe profile	[59].
	*C. novyi*-NT	Refractory solid tumors	Phase I	41% Decrease in the size of the injected tumor	[60]
Cells	Autologous dendritic cells	Melanoma; breast	Clinical study	Regression of the injected tumors	[61]
	Ilixadencel	Gastrointestinal stromal tumor	Phase I	33% Tumor responses	[62]
	Tumor-infiltrating lymphocytes	Melanoma	Clinical study	Tumor regression	[63]
	Erbb-targeted CAR-T	Squamous cell carcinoma	Phase I	60% Stabilization of disease	[64]
	C-Met-CAR-T	Breast	Phase 0	Evoke an inflammatory response within tumors	[65]
	Xenogeneic tissue cells	Bladder; breast; pancreatic	Preclinical murine model	Suppress tumor growth	[66,67]
